# Effect of p53 activation through targeting MDM2/MDM4 heterodimer on T regulatory and effector cells in the peripheral blood of Type 1 diabetes patients

**DOI:** 10.1371/journal.pone.0228296

**Published:** 2020-01-29

**Authors:** Marsha Pellegrino, Gianandrea Traversi, Andrea Arena, Marco Cappa, M. Manuela Rosado, Marco Andreani, Domenico V. Delfino, Fabiola Moretti, Alessandra Fierabracci

**Affiliations:** 1 Infectivology and Clinical Trials Research Department, Bambino Gesù Children’s Hospital, IRCCS, Rome, Italy; 2 Endocrinology Department, Bambino Gesù Children’s Hospital, IRCCS, Rome, Italy; 3 Research Laboratories, Bambino Gesù Children’s Hospital, IRCCS, Rome, Italy; 4 Transplantation Immunogenetics Laboratory, Bambino Gesù Children’s Hospital, IRCCS, Rome, Italy; 5 Section of Pharmacology, Department of Medicine, University of Perugia, Perugia, Italy; 6 Institute of Cell Biology and Neurobiology, National Research Council of Italy (CNR), Rome, Italy; Mississippi State University, UNITED STATES

## Abstract

Various immunotherapies for the treatment of type 1 diabetes are currently under investigation. Some of these aim to rescue the remaining beta cells from autoimmune attack caused by the disease. Among the strategies employed, p53 has been envisaged as a possible target for immunomodulation. We studied the possible effect of p53 activation on Treg subsets and Treg/Teff balance in type 1 diabetes patients’ PBMC. Upon p53 activation, we observed an increase in CD8+ Treg and activated CD8+ Teff whilst CD8+ Teff cells significantly decreased in healthy PBMC when stimulated with anti-CD3/CD28. No effect was detected on percentages of CD4+ Treg, while a reduction was seen in CD4+ Teff cells and an increase in activated CD4+ Teff cells. In patients’ PBMC, upon p53 activation followed by 6 days of anti-CD3/CD28 stimulation, CD8+ Treg and activated CD8+ Teff were increased while CD8+ Teff were decreased. No differences were detected in the CD4+ counterparts. CD8+ Teff PD1+, CD8+ Teff PD1low were increased upon p53 activation in type 1 diabetics compared to controls while CD8+ Teff PD1high were increased in both groups. The same increased percentages were detected for CD4+ counterparts. CD4+ Treg PD1high cells were decreased in diabetics upon p53 activation at day 6 of anti-CD3/CD28 stimulation. In conclusion, a Teff dysregulation is observed upon p53 activation suggesting that molecules promoting p53 cannot be used for therapy in type 1 diabetics.

## Introduction

Type 1 diabetes is an organ-specific autoimmune disease, where autoreactive T lymphocytes destroy β cells in the pancreatic islets which produce insulin [1,2]. Epidemiological studies estimate that the prevalence of diabetes has been increasing over the past 30–40 years [3]. The daily substitutive administration of repeated injections of insulin acts to save the patient from certain death but does not cure the ongoing autoimmune process [4]. The scientific community has therefore advanced the hypothesis that immunotherapies could halt the pathogenic mechanisms, preserving the residual hormone-producing cells and improving the metabolic stability of disease [5].

Several immunotherapeutic approaches have been tried in type 1 diabetes. However, despite the many trials carried out, insulin-independence in diabetic patients was not achieved [6]. In recent years, TRP53 (transformation-related protein 53) has been considered as a possible target for immune control based on its reactivation [6]. Indeed, p53 is part of a sequence-specific short-lived transcription factor with important genomic regulatory functions (reviewed (rev) in [6,7,8]). It is well known that in human cancer p53 mediates tumor suppression (rev in [6]). The responses activated by the protein include cell cycle arrest, cell programs of apoptosis, autophagy, senescence, metabolism, fertility and stem cell regulation (rev in [6]).

P53 presents two main regulators, MDM2 (mouse double minute 2) and MDM4 proteins. MDM2 is an ubiquitin ligase that can bind and ubiquitinate p53 for proteasome dependent degradation; however, its activity is more efficient when bound to MDM4, to form a heterodimer. P53 binding to MDM2 and MDM4 heterodimer strictly controls its function in normal cells [9,10]. Whereas p53 activation is obtained when it is detached from MDM4 and MDM2 and in some circumstances from the dissociation of the heterodimer.

The mechanism underlying p53-mediated suppression of autoimmunity remains to be precisely determined since the net effect of its activity as a transcription factor results from its balanced expression in immune as well as non-immune cells, such as the pancreatic islets. P53 mediates transcription of the *FOXP3* (Forkhead box protein 3) gene, which encodes for a master regulator of T regulatory cells (Treg) [11]. Accordingly, p53 influences Treg development and maintenance rather than interfering with Treg suppressive function [12].

The role of p53 in inflammation has been extensively investigated in animal models of autoimmunity [11–14] since systemic p53-deficient (p53^-/-^) mice experience a more rapid development of collagen- and antigen-induced arthritis (CIA) [15,16]. Furthermore, p53^-/-^ C57BL/6 mice treated with low-dose streptozotocin (STZ) showed a higher rate of type 1 diabetes incidence and higher circulating levels of proinflammatory cytokines [17]. Aged p53/cKO (conditional knockout) mice spontaneously developed several inflammatory diseases, in parallel to a reduction of CD4+ CD25+FOXP3+ Treg cells [12]. In DBA/1J and C57BL/6 p53^-/-^ mice affected by CIA, CD4+ T cells decreased the activity of STAT-5 (signal transducer and activator of transcription 5), lowering its protein levels, and compromised Treg cell differentiation [11]. Furthermore, CD4+ T cells from p53^-/-^ mice showed lower capacity to differentiate toward Treg cells, expressing lower FOXP3 levels. Administration of a p53 overexpression vector or an antagonist of MDM2 was able to control CIA development in mice [11]. Consistently, rheumatoid arthritis (RA) patients show lower p53 mRNA levels and higher percentages of circulating T helper 17 (Th17) cells compared to healthy controls. Thus, in these patients, inhibition of p53-MDM2 interaction by nutlin-3a induced Treg differentiation under Th17 polarizing conditions [11]. Moreover, FOXP3 expression increased significantly under these culture conditions. Consistent with p53 putative regulatory effect in autoimmunity, autoantibodies (AAbs) to the C-terminal domain of p53 were detected in the sera of patients with several autoimmune conditions [18–23] and these AAbs were shown to affect p53 protein function [18–23]. Thirty p53 target genes were detected in patients with benign multiple sclerosis [24]. *P53* gene mutations were discovered in human RA synoviocytes [25–27], a TP53 codon 72 *Arg/*Arg polymorphism was found associated with a higher risk for inflammatory bowel disease development [28] and autoimmune thyroid disease [29,30] and detected at high prevalence in type 1 diabetes patients with age at onset <6 years with a strong linear correlation in females [31].

In the light of the foregoing p53-targeted therapies, p53 activators, such as Nutlin-3a, have been considered as a potential therapeutic strategy in autoimmune diseases [6]. In this study, we aimed to investigate the possible effect of p53 reactivation on expansion of CD4+ and CD8+ Treg subsets and their balance with T effector cells in type 1 diabetes patients. Of note, the mechanism underlying p53 activation that will be used in this work is based on the use of an MDM2/MDM4 heterodimer inhibitor [32] and has already been extensively studied in human cancer cells [32].

## Materials and methods

### Subjects

The patients analyzed were 16 long-standing (long-term, LT) type 1 diabetes patients. LT subjects have at least 10 years of disease. Patients were enrolled at the Department of Endocrinology at Bambino Gesù Children’s Hospital (OPBG) over the past five years. Sera from patients were analyzed for the presence of diabetes-related autoantibodies (AAbs), i.e. glutamic acid decarboxylase isoform 65 (GADA), protein tyrosine phosphatase insulinoma-associated antigen 2 (IA2) and insulin (IAA) AAbs by radioimmunoassay (RIA); thyroglobulin (Tg), thyroperoxidase (TPO) and tissue transglutaminase (tTGA) AAbs were tested by chemiluminescence (ADVIA Centaur analyzer: Siemens Healthcare, Germany), parietal cell (PCA), adrenal cortex (ACA) and islet cell AAbs by indirect immunofluorescence (IFL). Mean glycated hemoglobin (HbA1c) value of patients was 61.31 mmol/mol for LT subjects (cut-off value 48 mmol/mol). C-peptide levels were below the normal range (0.80–3.80 ng/mL) indicating a poor metabolic control, thus requiring insulin dose adjustments. Overall insulin requirement was in the normal range for age. Patients had not developed any diabetes complication at the time of observation. The control group of 20 healthy donors (HD), without any family history of autoimmune disease and no circulating AAbs, was recruited from the OPBG Blood Transfusion Division. All controls were matched to patients for sex, age, ethnic and geographical origin. Recruited patients and controls were unrelated. All subjects enrolled in the investigation provided prior written informed consent in accordance with the Declaration of Helsinki. The study was approved by the local Institutional Review Board (IRB) of the OPBG, which regulates the use of human samples for experimental studies. Written informed consent for the children was obtained from their next of kin.

### Detection of p53 codon 72 polymorphism in type 1 diabetic patients

Genomic leukocyte DNA was extracted from whole blood samples of patients by QIAmp DNA blood mini kit (Qiagen, Hilden Germany) according to manufacturer’s guidelines. PCR (polymerase chain reaction) was carried out with specific primers for exon 4 of the gene (Gene Bank ID:7157): forward 5’-AATGGATGATTGATGCTGTCCC-3’ and reverse 5’ GGTGCAAGTCACAGACTTGGC-3’ [31]. The amplification lasted 35 cycles with 62°C annealing temperature. PCR sequencing was carried out with the BigDye Terminator v.3.1 Cycle sequencing protocol (Life Technologies, Applied Biosystems, Paisley, Scotland, UK). Products were then purified and sequenced with the Genetic Analyzer 3500 (Applied Biosystems HITACHI system).

### HLA typing

Patients were typed for HLA-A, -B, -C, -DRB1 and DQB1 loci at high resolution by PCR sequence specific oligonucleotide probes (PCR-SSO) using a commercial kit (LABType XR, One Lambda Inc. Canoga Park, CA) on a Luminex platform.

### Cell preparation

Ficoll-Hypaque protocol (Histopaque, Sigma-Aldrich Chemical: St Louis, MO, USA) was used to isolate peripheral blood mononuclear cells (PBMC) from sodium heparinized venous blood samples (5–10 mL). Subsequently, the samples were cryopreserved in liquid-nitrogen according to standard procedures [33,34].

### Treatment and stimulation of PBMC

Healthy donors and type 1 diabetes patients’ liquid-nitrogen frozen peripheral blood mononuclear cells (PBMC) were thawed in complete RPMI medium (GibcoTM RPMI 1640 Medium, ThermoFisher Scientific, Waltham, MA, USA) supplemented with 10% fetal bovine serum (FBS, Hyclone, South Logan, UT, USA), L-glutamine (2mM) (EuroClone S.p.A., MI, Italy) and 1% penicillin/streptomycin (pen/strep) (EuroClone) according to established protocols [34]. Cells were centrifuged at 1200 rpm for 5 minutes at room temperature (RT) and cultured in 48 well plates (Falcon, Corning 7 Incorporated, NY, USA), 1.5×10^6^ cells per well. Subsequently the cells were pre-treated at the indicated peptide 3 (Pep3) or peptide 3 mutated (Pep3 MUT) concentrations as previously described [32] for 24 hours (hrs). Pep3, composed of 12 aminoacids [32] was designed to selectively impair the MDM2/MDM4 heterodimer in order to release the inhibition on p53 so as to activate its function. Pep3 MUT has the same sequence of Pep3 except for a mutation in the key contact point of the peptide. This mutation in Pep3 MUT makes this peptide unable to impair the heterodimer formation, thus it represents Pep3 specificity control for the experiment. The following day, Dynabeads Human T-activator CD3/CD28 (Life Technologies AS, Oslo, Norway) were administered to stimulate cells at a bead-to-cell ratio of 1:50. This suboptimal anti-CD3/CD8 bead-to-cell ratio better reflects physiological conditions and allows detection of immunomodulatory activity [35]. Incubation of cells lasted four and six days at 37°C in a humidified atmosphere containing 5% CO_2_. At the end of the incubation period, cells were harvested from culture plates and washed by centrifugation 1200 rpm for 5 minutes in phosphate-buffered saline (PBS) at RT. Subsequently cells were stained for FACS analysis as described below.

### Flow cytometry analysis (FACS)

To analyze T cell subsets, cells were stained as already described [36] for 20 minutes at 4°C for surface markers detection. The antibodies used are listed as follows: Brilliant Ultraviolet 737 (BUV737) conjugated mouse anti-human CD3 (Clone UCHT1; 1:40 dilution; BD Biosciences, CA, USA); Brilliant Violet 421 (BV421) conjugated mouse anti-human CD25 (Clone M-A251; 1:40 dilution; BD); allophycocyanin (APC) conjugated mouse anti-human CD8 (Clone RPA-T8; 1:10 dilution; BD) and R-phycoerythrin-Cyanine7 (PE-Cy7) conjugated mouse anti-human CD279 (programmed cell death 1, PD1) (Clone J105; 1:40 dilution; eBioscience, ThermoFisher Scientific). After incubation, cells were washed once in PBS (EuroClone) 2% FBS and centrifuged at 1200 rpm for 5 minutes at RT. Subsequently, cells underwent FOXP3 intracellular staining using PE conjugated mouse anti-human FOXP3 antibody (Clone 259D/C7, BD) following manufacturer’s protocol (Human FOXP3 Buffer Set, BD). According to literature, CD8+ CD25+FOXP3+ cells were considered as CD8+ Treg and CD8+ CD25-FOXP3- cells as CD8+ effector T cells (Teff) [37,38]. CD8+ CD25+FOXP3- cells were considered activated CD8+ Teff cells. PD1+ cells within the total gate of CD8+ Treg and Teff were identified as CD8+ Treg PD1+ cells and CD8+ Teff PD1+ cells. Moreover, in this study the same populations as described above were also evaluated among the CD4+ subset (S1 Fig). Data were acquired using a Fortessa X-20 flow-cytometer (Becton and Dickinson (BD), Sunnyvale, CA, USA) and analyzed by FACSDiva software (BD Biosciences: San Jose, CA, USA). Dead cells were excluded from the analysis by side/forward scatter gating (S1 Fig). The acquisitions involved fifty thousand lymphocytes per sample.

### RNA extraction and quantitative real time-PCR analysis

Total RNA was isolated from HD and LT type 1 diabetes cryopreserved samples with TRIzol^™^ Reagent (Invitrogen, Life Technologies Corporation, Carlsbad, CA, USA) according to the manufacturer’s instructions. After in vitro reverse transcription (500 ng) with the High-Capacity cDNA reverse transcription kit (Applied Biosystems, Foster City, CA), quantitative Real-Time PCR (rtq-PCR) was performed using 7900HT Fast Real-Time PCR System (Applied Biosystems) and Power SYBR Green PCR Master Mix (Applied Biosystems). The following primers were used:

1. GAPDH (glyceraldehyde-3-phosphate dehydrogenase) (human): forward (F) 5’-CGACCACTTTGTCAAGCTCA-3’, reverse (R) 5’-AGGGGTCTACATGGCAACTG-3’.
2. p53 (human) F 5’-GTCTGGGCTTCTTGCATTCT-3’, R 5’-AATCAACCCACAGCTGCAC-3’ [39]. mRNA levels, normalized to GAPDH, were calculated as follows: 2-[ΔCt(Lipo/siRNA)-ΔCt(RPMI)] = 2-ΔΔCt, where ΔCt = Ct (p53)-Ct (GAPDH).

### Statistical analysis

Statistical significance was evaluated among the different cell conditions and treatments for all subsets analyzed in 14 HD and 16 type 1 diabetes (D) patients using Wilcoxon matched-pairs signed rank test. Differences in the expression of PD1 molecules in terms of percentages of PD1+ cells among subsets were studied using the Wilcoxon test. The analysis was carried out with the GraphPad Prism software version number 5.00 (GraphPad Software: San Diego, CA, USA). A result of p < 0.05 was considered statistically significant.

## Results

### Study population

Within the group of LT (long-term) type 1 diabetes (D) patients in the present study, the mean age was 25.8 years (ranging from 21 to 31 years; 8 males, 8 females) and the mean duration of the disease 15.5 years (ranging from 12 to 20 years). The mean age of the HD (healthy donor) controls was 23 years (ranging from 18 to 30 years). Demographic, clinical and laboratory characteristics of patients are shown in Table 1 and S1 Table. In addition to type 1 diabetes, two patients also developed autoimmune thyroiditis (AT, autoimmune polyglandular syndrome Type 3 variant, APS3v); one of the two was affected by Hashimoto’s thyroiditis confirmed by the presence of circulating Tg and TPO AAbs and echography pattern of diffuse hypoechogenicity. One patient developed Basedow’s disease (Table 1). *p53* *Arg/*Pro polymorphism was present in four patients, the remaining 12 harboring the *Pro/*Pro genotype. All harbored wild type (C1858C) *PTPN22* (S1 Table). Molecular typing results for HLA-A, -B, -C, -DRB1 and–DQB1 loci are shown in S2 Table.

**Table 1:** Demographic and clinical characteristics and autoantibody levels of the LT type 1 diabetes patients recruited for the study.

Pt	Sex	Age of Disease Onset (years)	Actual Age (years)	Disease Duration at referral (years)	Associated Diseases	Islet Related AAbs	Other AAbs
1	F	6	24	15		GADA 0.2 U/mL; IAA **44**%; IA2 0.1 U/mL	TPO< 28.0 U/mL; Tg<20.0 UI/mL; tTGA:1 CU
2	F	9	25	13	AT	GADA 0.1 U/mL; IA2 0.4 U/mL	TPO**>1300 U/mL**; Tg<20.0 UI/mL; tTGA:16.2 CU
3	F	14	31	13		GADA 0.6 U/mL; IAA 5.2%; IA2 0.7 U/mL	TPO:37.3 U/mL; Tg<20.0 UI/mL; tTGA:0.4 CU
4	M	5	23	14		GADA 0.3 U/mL; IA2 **4** U/mL	TPO<28.0 U/mL; Tg<20.0 UI/mL; tTGA:0.8 CU
5	M	3	25	19	Basedow	GADA 0.1 U/mL; IAA **14%**; IA2:0.6	TPO<28.0 U/mL; Tg<20.0 UI/mL; tTGA:16.3 CU
6	F	5	22	13		GADA 0.3 U/mL; IAA **10**%; IA2 0.1 U/mL	TPO<28.0 U/mL; Tg<20.0 UI/mL; tTGA:7.6 CU
7	M	7	27	17		GADA 0.2 U/mL; IAA **35**%; IA2 0.4 U/mL	TPO<28.0 U/mL; Tg<20.0 UI/mL; tTGA< 1.9 CU
8	M	2	24	20		GADA **2** U/mL; IA2 0.2 U/mL	TPO<28.0; Tg<20.0 UI/ml; tTGA:4 CU
9	M	9	30	18		GADA 0.3 U/mL; IA2:0.2 U/mL	TPO<28.0 U/mL; Tg<20.0 UI/ml; tTGA:4.4 CU
10	M	4	25	17		GADA 0.1 U/mL; IA2 **1** U/mL	TPO<28.0 U/mL; Tg<20.0 UI/ml; tTGA<1.0 CU
11	M	3	24	18		GADA 0.1 U/mL; IA2 0.2 U/mL	TPO<28.0 U/mL; Tg<20.0 UI/ml; tTGA<1.9 CU
12	F	10	31	17		GADA 0.6 U/mL; IA2 0.2 U/mL	TPO<28.0 U/mL; Tg<20.0 UI/ml; tTGA<1.9 CU
13	M	13	30	13		GADA 0.1 U/mL; IA2 0.2 U/mL	TPO<28.0 U/mL; Tg<20.0 UI/ml; tTGA<1.9 CU
14	F	9	26	13		GADA 0.2 U/mL; IA2 **15** U/mL	TPO**>1300** U/mL; Tg<20.0 UI/ml; tTGA:17.2 CU
15	F	6	22	12		GADA **5** U/mL; IA2 0.6 U/mL	TPO<28.0 U/mL; Tg<20.0 UI/ml; tTGA<1.9 CU
16	F	4	21	17		GADA 0.1 U/mL; IA2 0.1 U/mL	TPO<28; Tg<20.0 UI/ml; tTGA:0.8 CU

Islet- related autoantibodies (AAbs) reference values: glutamic acid decarboxylase isoform 65 (GADA) <0.90 Units/milliliter (U/mL); protein tyrosine phosphatase insulinoma-associated antigen 2 (IA2) < 1.1 U/mL and insulin (IAA) < 6.40%; other AAbs reference values: thyroperoxidase (TPO) < 60 U/mL; thyroglobulin (Tg) 0–40 UI/mL; tissue transglutaminase (tTGA): < 20 CU. Pt = patient. AT, autoimmune thyroid disease.

### Peptide 3 efficacy on HD PBMC subsets

**Fig 1:**
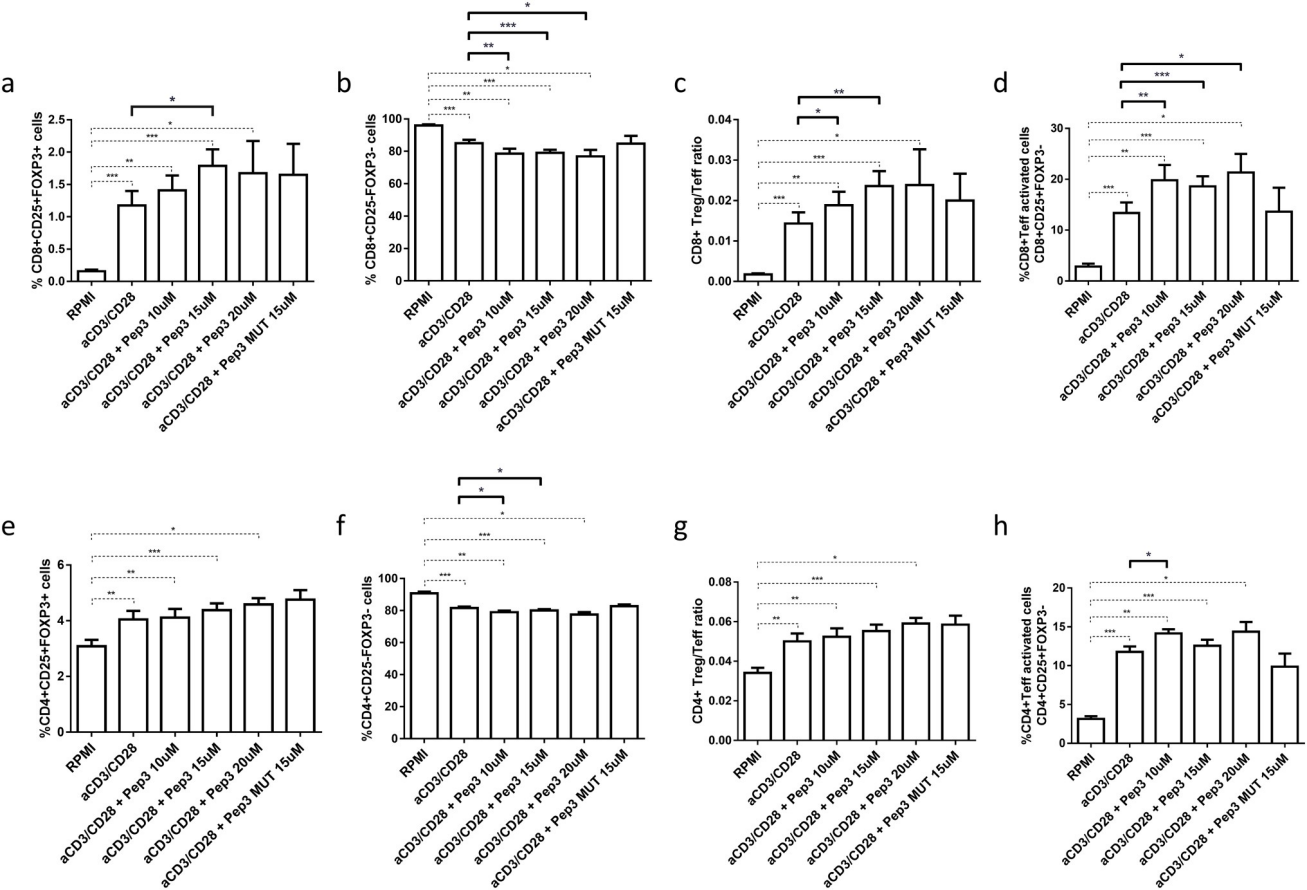
Frequency of T cell populations in PBMC isolated from healthy donors (HD) after treatment with peptide 3 and subsequent stimulation with anti-CD3/CD28 beads for 4 days. Flow-cytometry analysis of HD PBMC untreated unstimulated (RPMI) or stimulated (anti-CD3/CD28), treated with 10, 15 and 20μM Pep 3 or 15μM of Pep3 MUT and subsequently stimulated with anti-CD3/CD28 beads for four days. Percentages of CD8+ subsets (CD8+ Treg, CD8+ Teff, CD8+ Teff activated) were calculated on total CD8+ cells. Percentages of CD4+ subsets (CD4+ Treg, CD4+ Teff, CD4+ Teff activated) were calculated on total CD4+ cells. Percentages of total CD8+ and CD4+ cells were calculated on total lymphocytes. Graphs show the percentage of CD8+ Treg as CD8+ CD25+FOXP3+ cells (**a**), CD8+ Teff as CD8+ CD25-FOXP3- cells (**b**), CD8+ Treg/Teff ratio (**c**), CD8+ Teff activated cells as CD8+ CD25+FOXP3- cells (**d**), CD4+ Treg as CD4+ CD25+FOXP3+ cells (**e**), CD4+ Teff as CD4+ CD25-FOXP3- cells (**f**) (please refer for gating strategy to S1 Fig), CD4+ Treg/Teff ratio (**g**), CD4+ Teff activated cells as CD4+ CD25+FOXP3- cells (**h**). Values correspond to mean frequency ± SEM of 14 HD. * p<0.05, ** p<0.01, *** p<0.001.

In HD PBMC pre-treated with Pep3 and subsequently stimulated for 4 days with anti-CD3/CD28 beads, the percentage of CD8+ Treg significantly increased upon Pep3 treatment in a dose dependent manner between 10 and 15μM (Fig 1a; S2 Fig) compared to untreated stimulated cells. The percentage of CD8+ Teff decreased significantly upon the same peptide treatment (Fig 1b); consequently, the ratio between the two subsets increased independently of Pep3 concentration (Fig 1c). Notably, the CD8+ Teff activated cell subset (CD8+CD25+FOXP3- cells) increased significantly upon stimulation and the same Pep3 concentrations (Fig 1d). It is noteworthy that no significant change was detected in cultures pretreated with the highest dose, 15μM, of mutated peptide 3 (Pep3 MUT), indicating specificity of Pep3 activity (Fig 1a–1d). Regarding CD4+ Treg cells, under the same conditions no significant modulations were observed after Pep3 treatment (Fig 1e) while a significant decrease was detected in CD4+ Teff cells upon 10 and 15μM of treatment (Fig 1f). The CD4+ Treg/Teff ratio was not affected by the Pep3 treatment (Fig 1g). As observed above, CD4+ Teff activated cells (CD4+CD25+FOXP3- cells) significantly increased in percentage upon 15μM of peptide 3 (Fig 1h). No significant change was detected in the cultures treated with 15μM of the mutated peptide 3 (Pep3 MUT) (Fig 1e–1h). These results show that in PBMC of healthy individuals, Pep3 treatment, previously shown to induce p53 activity [32], can alter the percentages of CD8+ Treg, CD8+ and CD4+ Teff cells as well as of CD8+ and CD4+ activated Teff cells.

**Fig 2:**
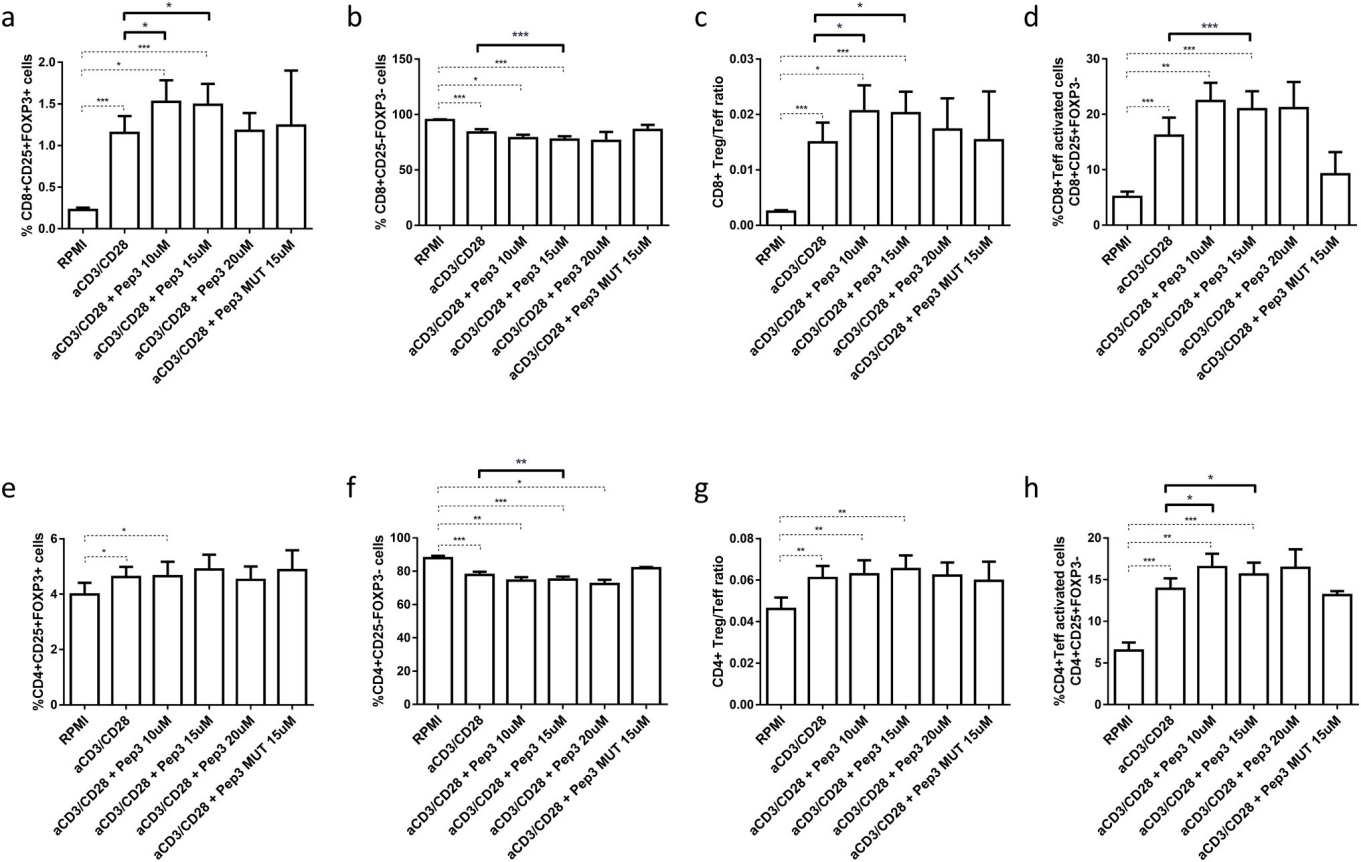
Frequency of T cell populations in PBMC from HD upon treatment with peptide 3 and subsequent stimulation with anti-CD3/CD28 beads for 6 days. Flow-cytometry analysis of HD PBMC following 10, 15 and 20μM treatment relative to Pep3 and 15μM treatment relative to Pep3 MUT and subsequent stimulation for six days with anti-CD3/CD28 beads. Graphs show the percentage of CD8+ Treg as CD8+ CD25+FOXP3+ cells (**a**), CD8+ Teff as CD8+ CD25-FOXP3- cells (**b**), CD8+ Treg/Teff ratio (**c**), CD8+ Teff activated cells as CD8+ CD25+FOXP3- cells (**d**), CD4+ Treg as CD4+ CD25+FOXP3+ cells (**e**), CD4+ Teff as CD4+ CD25-FOXP3- cells (**f**), CD4+ Treg/Teff ratio (**g**), CD4+ Teff activated cells as CD4+ CD25+FOXP3- cells (**h**). Percentages were obtained as described for the previous experiments (see legend to Fig 1). Values correspond to mean frequency ± SEM of 14 HD. * p<0.05, ** p<0.01, *** p<0.001.

After 6 days stimulation with anti-CD3/CD28 beads, similar results were observed. Indeed, compared to control untreated stimulated cells, percentages of CD8+ Treg increased significantly upon 10 and 15μM of peptide 3 (Fig 2a) treatment whereas CD8+ Teff cells decreased (Fig 2b) resulting in an increased ratio between the two subsets at both Pep3 concentrations (Fig 2c). Again, CD8+ Teff activated cells increased in respect to stimulated cells with 15μM of treatment (Fig 2d). No significant modulations were observed for CD4+ Treg after 6 days of stimuli (Fig 2e), while percentages of CD4+ Teff showed a significant decrease with 15μM of Pep3 treatment (Fig 2f); thus, the corresponding ratio to Treg cells was not affected by any concentration (Fig 2g). Of note, percentages of CD4+ Teff activated cells augmented upon 10 and 15μM of treatment (Fig 2h). For every cell subset analyzed, cultures pre-treated with 20μM of Pep3 or 15μM of control-mutated peptide did not exhibit any alteration as compared to control cultures (Fig 2).

Overall, these results show that after a prolonged period of culture a significant effect is maintained in CD8+Treg, CD8+ Teff, CD8+ activated Teff and CD4+ Teff while percentages of CD4+ activated T cells further increased.

### Peptide 3 efficacy on type 1 diabetes PBMC subsets

**Fig 3:**
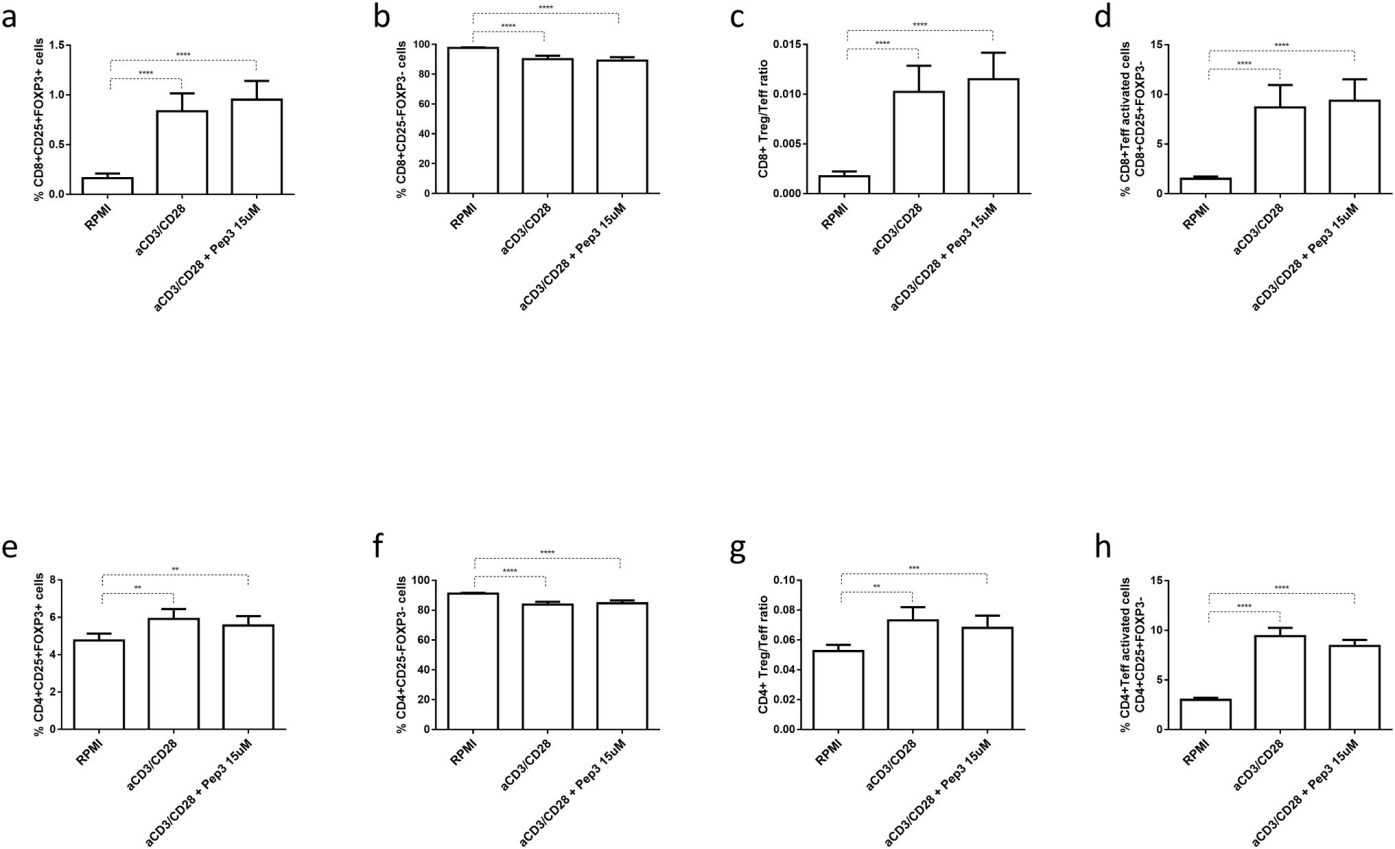
Frequency of T cell populations in PBMC isolated from type 1 diabetes patients treated with peptide 3 and subsequently stimulated with anti-CD3/CD28 beads for 4 days. Flow-cytometry analysis of type 1 diabetes PBMC non-treated non-stimulated (RPMI) or stimulated (anti-CD3/CD28), treated with 15μM Pep3 and subsequently stimulated with anti-CD3/CD28 beads for four days. Percentages were obtained as described for the previous experiments (see legend to Fig 1). Graphs show the percentage of CD8+ Treg as CD8+ CD25+FOXP3+ cells (**a**), CD8+ Teff as CD8+ CD25-FOXP3- cells (**b**), CD8+ Treg/Teff ratio (**c**), CD8+ Teff activated cells as CD8+ CD25+FOXP3- cells (**d**), CD4+ Treg as CD4+ CD25+FOXP3+ cells (**e**), CD4+ Teff as CD4+ CD25-FOXP3- cells (**f**), CD4+ Treg/Teff ratio (**g**), CD4+ Teff activated cells as CD4+ CD25+FOXP3- cells (**h**). Values correspond to mean frequency ± SEM of 16 LT type 1 diabetes patients. ** p<0.01, *** p<0.001, **** p<0.0001.

We next evaluated the effect of the peptide under study on PBMC from type 1 diabetes patients. Since each sample contained a limited number of cells, we chose to pre-treat cells with only one concentration of Pep3, 15μM. After 4 days of stimulation, no significant modulation was observed in cultures pretreated with Pep3, compared to untreated stimulated cells, for any of the CD8+ (Fig 3a–3d) and CD4+ subsets analyzed (Fig 3e–3h).

**Fig 4:**
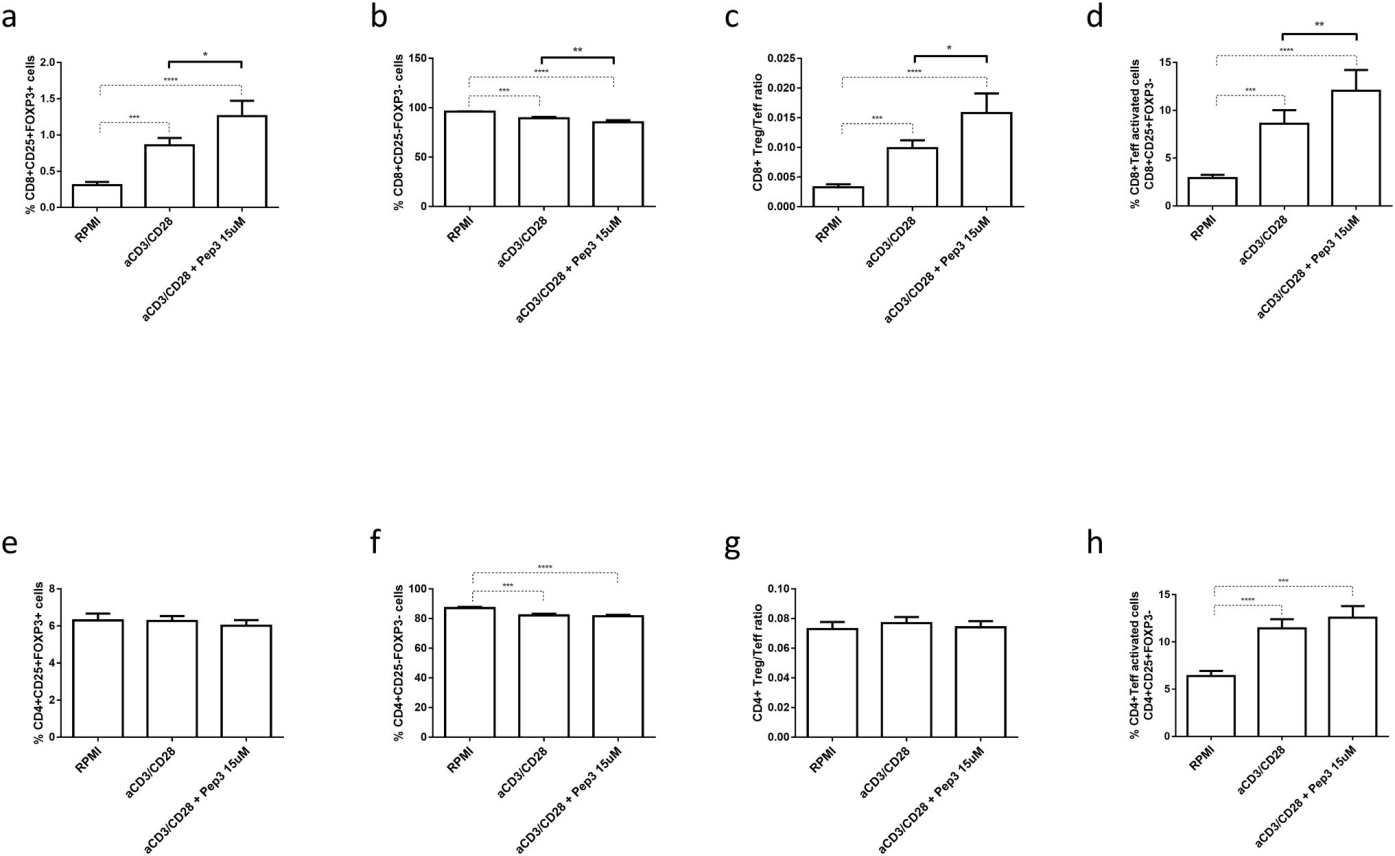
Frequency of T cell populations relative to type 1 diabetes PBMC upon treatment with peptide 3 and subsequent stimulation with anti-CD3/CD28 beads for 6 days. Flow-cytometry analysis of type 1 diabetes PBMC following Pep3 15μM treatment and subsequent stimulation for six days with anti-CD3/CD28 beads. Graphs show the percentage of CD8+ Treg as CD8+ CD25+FOXP3+ cells (**a**), CD8+ Teff as CD8+ CD25-FOXP3- cells (**b**), CD8+ Treg/Teff ratio (**c**), CD8+ Teff activated cells as CD8+ CD25+FOXP3- cells (**d**), CD4+ Treg as CD4+ CD25+FOXP3+ cells (**e**), CD4+ Teff as CD4+ CD25-FOXP3- cells (**f**), CD4+ Treg/Teff ratio (**g**), CD4+ Teff activated cells as CD4+ CD25+FOXP3- cells (**h**). Values correspond to mean frequency ± SEM of 16 LT type 1 diabetes patients. * p<0.05, ** p<0.01, *** p<0.001, **** p<0.0001. Percentages were obtained as described for the previous experiments (see legend to Fig 1).

Interestingly, after 6 days of anti-CD3/CD28 stimulation, compared to patient’s PBMC, the subsets analyzed showed an increased percentage of CD8+ Treg (Fig 4a), decrease in percentage of CD8+ Teff (Fig 4b) and a relative increment in the corresponding ratio of CD8+ Treg/Teff upon peptide 3 treatment (Fig 4c). Of note, the percentage of CD8+ Teff activated cells increased in cultures pre-treated with the peptide compared to untreated stimulated cells (Fig 4d). For all CD4+ subsets analyzed at 6 days post stimulation, Pep3 treatment showed no effect on the corresponding percentages (Fig 4e–4h).

These results indicate that Pep3 activity, which according to literature [32] induces p53 function, alters the percentages of CD8+ Treg, CD8+ Teff and CD8+ activated Teff in type 1 diabetes PBMC as well as in HD PBMC but with different kinetics. That is, the effects of p53 activation on CD8+ stimulated cell compartments are delayed in T1D patients. No effect is observed on CD4+ Treg and Teff subsets.

### P53 mRNA levels

A limited survey of p53 expression was conducted on PBMC of LT type 1 diabetes patients and of controls to compare the corresponding mRNA levels. No significant difference was observed in p53 mRNA levels, normalized to GAPDH, in patients compared to normal controls (Table 1, S3 Fig). This suggests that the different levels of p53 activity observed among patients is not related to alterations in basal p53 mRNA expression.

### Peptide 3 efficacy on the expression of PD1 molecule in HD and type 1 diabetes PBMC subsets

In order to investigate T cell activation following Pep3 treatment, in HD and type 1 diabetes PBMC, expression of the regulatory PD1 molecule was studied after 24 hrs treatment with the peptide and subsequent anti-CD3/CD28 stimulation.

**Fig 5:**
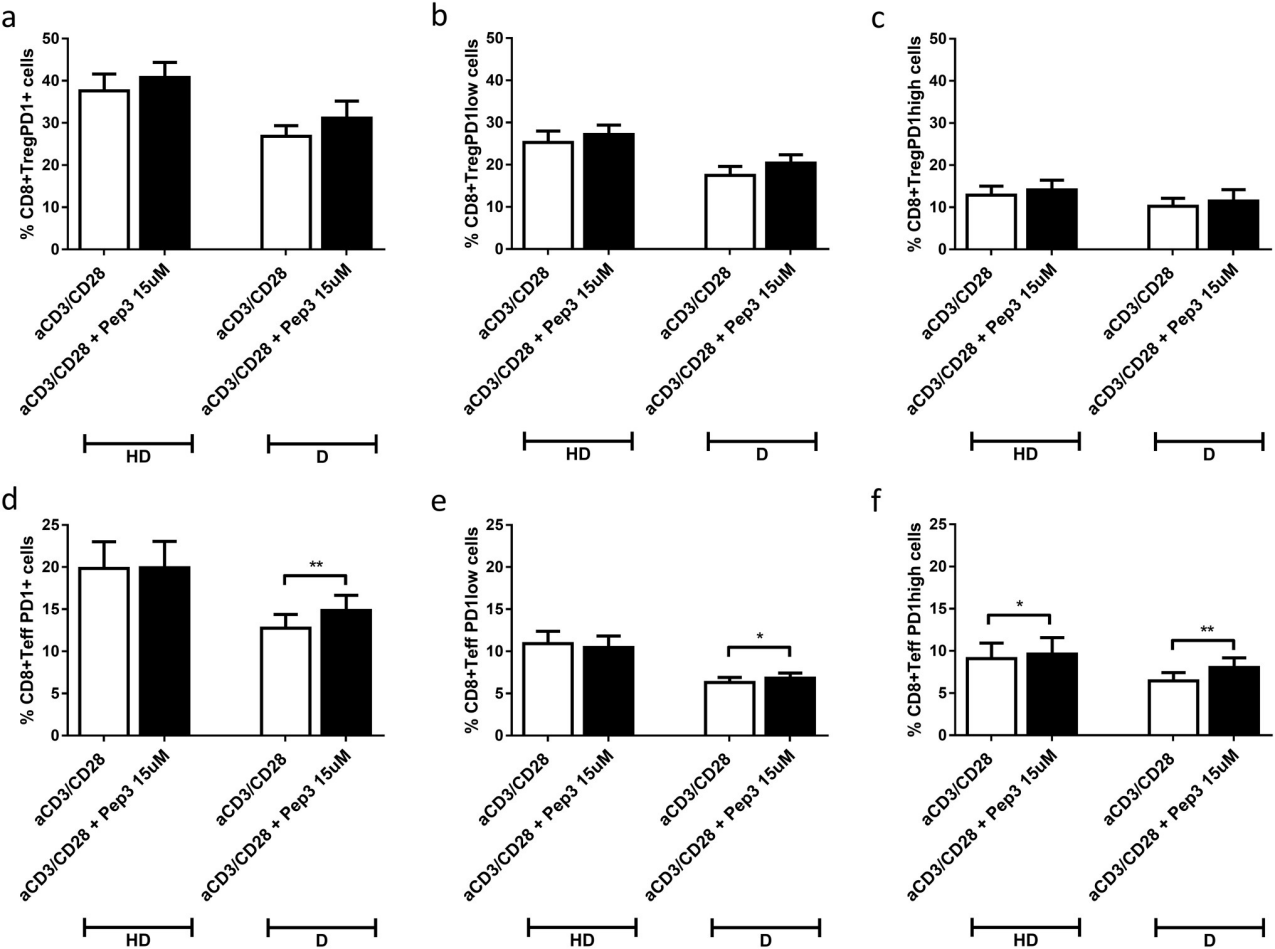
Frequency of CD8+PD1+ cell populations in PBMC of HD and type 1 diabetes upon treatment with peptide 3 and subsequent stimulation with anti-CD3/CD28 beads for 4 days. Flow-cytometry analysis of HD and type 1 diabetes PBMC following 15μM of Pep3 treatment and subsequent stimulation for four days with anti-CD3/CD28 beads. Graphs show the percentage of CD8+ Treg PD1+ cells (**a**), CD8+ Treg PD1low cells (**b**), CD8+ Treg PD1high cells (**c**), CD8+ Teff PD1+ cells (**d**), CD8+ Teff PD1low cells (**e**), CD8+ Teff PD1high cells (**f**). Percentages of PD1+, PD1low and PD1high cells were evaluated in comparison to the corresponding parental subset under evaluation. Values correspond to mean frequency ± SEM of 14 healthy controls (HD) and 16 long-term type 1 diabetes patients (D). * p<0.05, ** p<0.01.

After 4 days of stimulation, no significant difference was observed upon Pep3 administration on the percentages of CD8+ Treg PD1+ cells (Fig 5a), CD8+ Treg PD1low cells (Fig 5b) and CD8+ Treg PD1high cells (Fig 5c) in either HD or type 1 diabetes PBMC. Conversely, Pep3 administration led to a partial recovery of the levels of CD8+ TeffPD1+ cells in treated type 1 diabetes PBMC whereas it was ineffective in HD PBMC (Fig 5d). Similar results were observed for CD8+ Teff PD1low cells (Fig 5e). Percentages of CD8+ Teff PD1high cells were similarly increased by Pep3 in both HD and type 1 diabetes PBMC (Fig 5f). Comparable results were obtained after 6 days of stimulation (S4 Fig). Furthermore, no significant difference was observed upon Pep3 administration at either day 4 or day 6 of anti-CD3/CD28 stimulation for percentages of CD8+ Teff activated PD1+ cells (S5a and S5d Fig), for CD8+ Teff activated PD1low cells (S5b and S5e Fig) and for CD8+ Teff activated PD1high cells (S5c and S5f Fig) for both HD and type 1 diabetes PBMC. These results indicate a response of CD8+ Teff PD1+, CD8+ Teff PD1low and CD8+ Teff PD1 high subsets in type 1 diabetics due to their PBMC dysregulation compared to normal controls. Increased percentages of CD8+ Teff PD1 high were observed only in HD PBMC.

**Fig 6:**
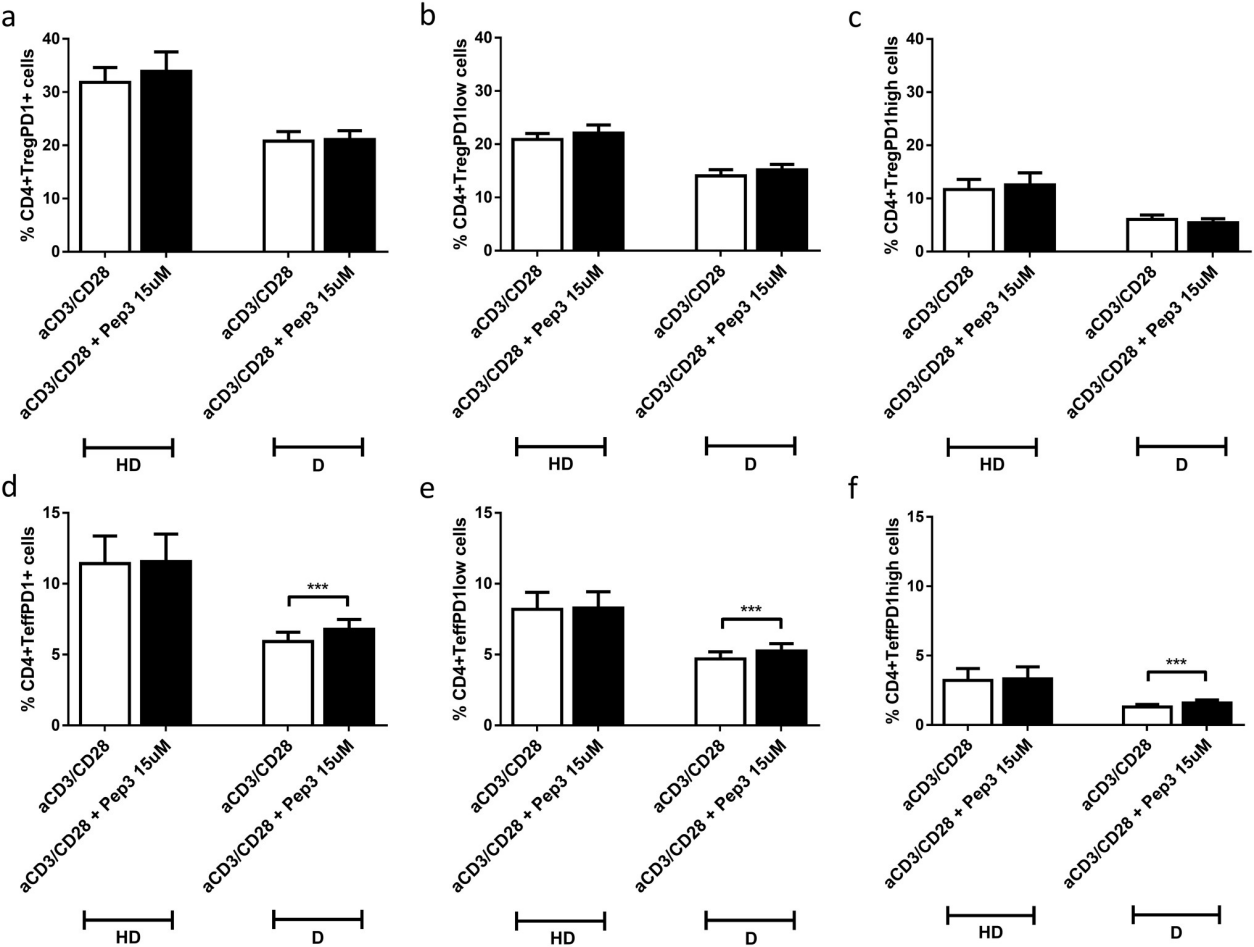
Frequency of CD4+PD1+ cell populations relative to HD and type 1 diabetes upon treatment with peptide 3 and subsequent stimulation with anti-CD3/CD28 beads for 4 days. Flow-cytometry analysis of HD and type 1 diabetes PBMC following 15μM of Pep3 treatment and subsequent stimulation for four days with anti-CD3/CD28 beads. Graphs show the percentage of CD4+ Treg PD1+ cells (**a**), CD4+ Treg PD1low cells (**b**), CD4+ Treg PD1high cells (**c**), CD4+ Teff PD-1+ cells (**d**), CD4+ Teff PD-1low cells (**e**), CD4+ Teff PD-1high cells (**f**). Percentages of PD1+, PD1low and PD1high cells were evaluated in comparison to the corresponding parental subset under evaluation. Values correspond to mean frequency ± SEM of 14 healthy controls (HD) and 16 long-term type 1 diabetes patients (D). *** p<0.001.

Percentages of CD4+ Treg PD1+ cells from both HD and type 1 diabetes PBMC pre-treated for 24 hrs with peptide 3 showed no significant difference after 4 days of anti-CD3/CD28 stimulation between untreated and treated cells (Fig 6a). Also, no difference was detected in percentages of CD4+ Treg PD1low and CD4+ Treg PD1 high cells for HD and type 1 diabetes patients (Fig 6b and 6c respectively). An increase was observed for the percentages of CD4+ Teff PD1+, CD4+ Teff PD1 low and CD4+ Teff PD1high cells of type 1 diabetes PBMC, post Pep3 administration, in respect to untreated cells (Fig 6d–6f). Similar modulations upon Pep3 treatment were observed after 6 days of stimulation (S6 Fig); increased percentages of CD4+ Teff PD1high cells were also observed in HD controls (S6f Fig). In addition, no significant difference was observed upon Pep3 administration at 4 days of anti-CD3/CD28 stimulation in percentages of CD4+ Teff activated PD1+ cells (S7a Fig), CD4+ Teff activated PD1 low cells (S7b Fig) and CD4+ Teff activated PD1high cells (S7c Fig) for both HD and type 1 diabetes PBMC. Upon Pep3 administration at day 6 of anti-CD3/CD28 stimulation no significant difference was observed in percentages of CD4+ Teff activated PD1+ cells (S7d Fig) and CD4+ Teff activated PD1low cells (S7e Fig) for both HD and type 1 diabetes PBMC. No significant difference was observed in percentages of CD4+ Teff activated PD1high cells in HD controls whilst a significant decrease was observed in type 1 diabetics (S7f Fig).

## Discussion

The hypothesis that the tumor suppressive factor p53 could have a potential role in controlling autoimmunity has been under consideration since the observation that p53-null mice present enlarged lymphoid compartments within the body [40]. Therefore, correcting a dysfunctional p53-regulatory pathway could also represent a new therapeutic option for autoimmune diseases or associated conditions of cancer and autoimmunity [6,41]. Small-molecule inhibitors of p53-MDM2 interaction are already being exploited for cancer treatment with synthetic drugs. Of these, Nutlin was the first to be investigated. Furthermore, Phase I clinical trials involving novel and advanced MDM2 inhibitors have been recently undertaken.

Different groups have shown that nutlin-3a is capable of controlling autoimmune arthritis in p53^+/+^ mice via STAT-mediated regulation of the Th17 cell/Treg cell balance [11] and via upregulation of Treg cells in human RA patients. These observations suggest that p53 is involved in the development/control of autoreactive T cells. Indeed, p53 may potentially suppress autoimmunity by promoting the transcription of FOXP3 in the Treg subset and inducing Treg cell differentiation. Unfortunately, Nutlin is known to exhibit strong toxic effects [42,43]. We therefore decided to employ a different p53 activating strategy, already validated in literature [32], with the aim of increasing Treg subsets and altering the Treg/Teff balance to obtain beneficial effects in type 1 diabetes patients.

In our experimental setup, we aimed to evaluate immunotypes in physiological conditions. Pep3, a small peptide known to selectively impair the MDM2/MDM4 heterodimer formation [6,32], was employed in order to release the inhibition on p53 and activate its function.

Upon indicated doses of Pep3 pre-treatment and 4 days of anti-CD3/CD28 stimulation in HD PBMC, percentages of CD8+ CD25+FOXP3+ Treg were significantly increased. Total CD8+ CD25-FOXP3- Teff cells were decreased and CD8+ CD25+FOXP3- Teff activated increased. Consistently, CD8+ Treg/Teff ratio was increased. Pep3-mediated activation of p53 had no effect on percentages of CD4+ CD25+FOXP3+ Treg; reduction of total CD4+CD25-FOXP3- was achieved while stimulation of CD4+ CD25+FOXP3- Teff activated was observed. Similar results were confirmed after 6 days of stimulation although HD PBMC showed a more pronounced effect on CD4+ CD25+FOXP3- Teff activation. After 6 days of stimulation and 15μM Pep3, percentages of type 1 diabetes patients’ CD8+ Treg were increased, total CD8+ Teff were diminished, CD8+ Treg/Teff ratio was increased as were the percentages of activated CD8+ Teff within the total CD8+ Teff. Regarding CD4+ counterparts, no significant differences were detected among the subsets analyzed.

The PD1/PDL1 (programmed death-ligand 1) pathway is known to affect the function of several immunotypes. In particular, it can influence Treg generation and suppressive functions, is implicated in the negative modulation of reactivation, expansion, and activity of effector T cells [44,45] and in their interactions with Treg [46]. P53 also exerts an effect on the PD1/PDL1 pathway regulation and expression; in particular, p53 activation by Nutlin-3 was shown to induce PD1 expression on cancer cells [47,48]. In this regard, a correlation of p53 with immune checkpoint status has been clearly indicated since the presence of p53 mutations (eventually associated with KRAS (Ki-ras2 Kirsten rat sarcoma viral oncogene homolog) mutant) in cancer patients has a potential predictive value on the response to anti-PD1 therapy [49].

It is interesting to note that in our study, upon Pep3 pre-treatment after 4 or 6 days of stimulation, percentages of total CD8+ Teff PD1+, CD8+ Teff PD1low PBMC were significantly increased in type 1 diabetes patients compared to normal controls, while percentages of CD8+ Teff PD1high were increased in both diabetics and normal controls. Of note, and consistent with this result, we have previously demonstrated a diminished expression of total PD1+, PD1high and PD1low Teff subsets in a subgroup of LT type 1 diabetes patients among those of the present investigation (patients 1–7 in Table 1) [32]. The same increased percentages were detected for total CD4+ Teff PD1+, CD4+ Teff PD1low and CD4+ Teff PD1high in the present study. Percentages of CD4+ Treg PD1 high cells were decreased in diabetic PBMC upon p53 activation at day 6 of anti-CD3/CD28 stimulation in diabetic PBMC.

In this respect, the use of checkpoint inhibitors was suggested. In particular, anti–PD1, and anti–PDL1 antibodies could be responsible for a rapid progression of autoimmune diabetes in human subjects with genetic predisposition to the disease [45,50]. The results obtained on the increased PD1 expression in CD8+ and CD4+ Teff cells imply a mechanistic effect of p53 activation on these lymphocytes [36]. Thus, we can speculate that PD1 expression induced by Pep3-dependent p53 activation functions as a compensatory mechanism to their expansion. Furthermore, consistent with immune dysregulation in type 1 diabetes, CD4+ Teff activated PD1high were decreased in type 1 diabetes patients at day 6 of anti-CD3/CD28 stimulation upon Pep3 pretreatment.

It is noteworthy that, in our study, responses to Pep3-dependent p53 activation were more evident for CD8+ Treg than for CD4+ Treg cells. We recently identified and characterized CD8+ CD25+FOXP3+ Treg cells in the peripheral blood of type 1 diabetes patients [36]. This regulatory subset was previously reported in mice and humans, but was not fully characterized since unique markers were unavailable. In the meantime, several markers of suppressive CD8+ Treg have been reported, such as the presence of CD25, CD56, CXCR3, FOXP3, CD122, CD38, CD8αα, CD45RA, CD45RO, LAG-3 and/or HLA-G, as well as the absence of CD28 and CD127 expression [36,37,51]. Because of the absence of CD28 reported for the CD8+ Treg subset [36], studies regarding their functional evaluation were conducted in our laboratory using the polyclonal phorbol-12-myristate-13-acetate (PMA)-ionomycin as T cell stimulator [36]. Concerning the present investigation, the response of CD8+ Treg cells to Pep3-dependent p53 activation was depicted in an experimental setup utilizing anti-CD3/CD28 stimulation of total PBMC [51]. This stimulus was chosen in several studies including our own [33] for CD4+ Treg activation and expansion. Although CD28+ Treg have been described as negative for CD28 expression, a study by Muthu Raja et al [52] described them as FOXP3+, CTLA-4+, CD45RO+, CD62L+ and CD28+ but not CD127+. Thus, the expression of CD28 by human CD8+ Treg cells remains controversial suggesting that the observed effect on the CD8+ Treg is indirect, caused by the anti-CD28 costimulation on other immunotypes.

These results clearly indicate that in type 1 diabetes patients, p53 activation by Pep3-dependent mechanism [32] increased the frequency of CD8+ Treg cells. However, this effect paralleled the increase of the Teff cell subset, particularly of the activated Teff cells. These experimental data obtained in vitro strongly suggests that this specific p53 activation cannot be recommended as an immunotherapeutic approach in type 1 diabetes patients, either at onset or in the long-term when a beta cell reservoir is still present, since it would promote the activation of Teff cells, which is definitely not beneficial for disease progression. This result is the opposite to that hypothesized in published literature [6] and in contrast to observations concerning the effect of Nutlin-mediated p53 reactivation in controlling arthritis development in p53^+/+^ mice [11]. Although the results were achieved using a different method of p53 reactivation and different experimental approaches, the discrepancy of the results of the two different models of autoimmune diseases may indicate that p53 activation cannot be used as an immunotherapeutic approach for all categories of autoimmune disorders. Furthermore, we may speculate that this treatment should be applied exclusively to conditions where there is a documented defect of wild-type p53 expression as observed by Park et al. [11] in the case of RA patients. Of note, in the present work, a limited survey of PBMC of LT patients and controls detected no significant difference in p53 mRNA levels. Also, only 4 out of the 16 type 1 diabetes patients enrolled harbored a p53 polymorphism which, however, did not affect p53 mRNA expression. Nonetheless, this finding does not exclude the possibility of an altered basal p53 activation in our cohort of type 1 diabetes patients [31] and suggests that different effects of p53 activation among patients and controls are not due to different p53 expression levels. As regard p53 pathways were indeed reported as upregulated in T1D [31]. Nevertheless, it is difficult to estimate whether at the time of sampling PBMC of patients in the present cohort could have been exposed over time to fluctuating levels of hyperglycemia able to induce p53 activation [53].

On a speculative basis, the p53 codon 72 polymorphism resides outside TAD domain (approximately residues 1–60 of p53) responsible for interaction with MDM2/MDM4 [54]. In particular, p53 employs a restricted region (approximately residues 16–30) for this interaction although the entire p53 TAD domain is necessary to modulate these interactions. In fact, under stress conditions, the TAD domain undergoes phosphorylation on several residues indirectly abrogating p53 binding affinity with interactors including MDM2/MDM4. However, codon 72 is outside either the region involved in the binding as well as the multiple sites undergoing phosphorylation. Furthermore, the codon 72 and flanking residues are poorly conserved even in orthologues of close species (mammals such as cat, mouse and cattle), which suggests that codon 72 and surrounding residues have no functional role in MDM2/4 binding or its regulation.

Nevertheless since p53 codon 72 polymorphism is a strong apoptosis inducer [55] we may suggest that under p53 activation followed by anti-CD3/CD28 stimulation Treg, Teff and activated Teff subsets may have increased susceptibility to apoptosis in PBMC of T1D patients harboring the variant. Analysis of an extended group of patients could help to ascertain this interesting point.

## Supporting information

S1 Fig Gating strategy. Flow cytometry profiles and gating strategy for the analysis of CD8+ and CD4+ T cell subsets. Data were acquired with flow-cytometer Fortessa X-20 analyzer (BD) and analyzed by FACSDiva software (BD Biosciences). Lymphocytes were gated through their forward/scatter properties (FSC-A×SSC-A plot). 50,000 lymphocytes were acquired. In this example, nitrogen frozen PBMC were thawed, stained as described in the method section for antibodies to CD3, CD8, CD25, PD1 and FOXP3, and subsequently analyzed. The plots show depiction of CD8+ subsets, CD8+ Treg and CD8+ Teff and CD8+ Teff activated and the analysis of PD1+, PD1high and PD1low, in RPMI (**a**) and after anti-CD3/CD28 stimulation (**b**); plots show also the CD4+subsets, CD4+ Treg and CD4+ Teff and CD4+ Teff activated and the analysis of PD1+, PD1high and PD1low, in RPMI (**c**) and after anti-CD3/CD28 stimulation (d).
(TIF)

S2 Fig Representative CD8+ Treg increase after Pep3 treatment. Images show representative plots indicating and comparing the percentages of CD8+ Treg among RPMI, anti-CD3/CD28 stimulated and anti-CD3/CD28 stimulated cells pretreated with 15μM of Pep3.
(TIF)

S3 Fig P53 mRNA levels in PBMCs. Messenger RNA for p53 in PBMC from 7 LT type 1 diabetic patients and 9 HD controls was quantified by rtq-PCR analysis. Each symbol represents an individual; horizontal lines show the mean ± SEM. p = 0.7377.
(TIF)

S4 Fig Frequency of CD8+PD1+ cell populations relative to HD and type 1 diabetes upon treatment with peptide 3 and subsequent stimulation with anti-CD3/CD28 beads for 6 days. Graphs show the percentage of CD8+ Treg PD1+ cells (**a**), CD8+ Treg PD1low cells (**b**), CD8+ Treg PD1high cells (**c**), CD8+ Teff PD1+ cells (**d**), CD8+ Teff PD1low cells (**e**), CD8+ Teff PD1high cells (**f**). Percentages of PD1+, PD1low and PD1high cells were evaluated in comparison to the corresponding parental subset under evaluation. Values correspond to mean frequency ± SEM of 14 healthy controls (HD) and 16 long-term type 1 diabetes patients (D). * p< 0,05 ** p<0,01.
(TIF)

S5 Fig Frequency of CD8+ activated PD1+ cells relative to HD and type 1 diabetes upon treatment with peptide 3 and subsequent stimulation with anti-CD3/CD28 beads. Upper graphs (**a**,**b**,**c**) show the percentage of CD8+ Teff activated PD1+ cells (**a**), CD8+ Teff activated PD1low cells (**b**), CD8+ Teff activated PD1high cells (**c**) after 4 days of anti-CD3/CD28 stimulation. Lower graphs (**d**,**e**,**f**) show the percentage of CD8+ Teff activated PD1+ cells (**d**), CD8+ Teff activated PD1low cells (**e**), CD8+ Teff activated PD1high cells (**f**) after 6 days of anti-CD3/CD28 stimulation Values correspond to mean frequency ± SEM of 14 healthy controls (HD) and 16 long-term type 1 diabetes patients (D).
(TIF)

S6 Fig Frequency of CD4+PD1+ cell populations relative to HD and type 1 diabetes upon treatment with peptide 3 and subsequent stimulation with anti-CD3/CD28 beads for 6 days. Graphs show the percentage of CD4+ Treg PD1+ cells (**a**), CD4+ Treg PD1low cells (**b**), CD4+ Treg PD1high cells (**c**), CD4+ Teff PD1+ cells (**d**), CD4+ Teff PD1low cells (**e**), CD4+ Teff PD1high cells (**f**). Values correspond to mean frequency ± SEM of 14 healthy controls (HD) and 16 long-term type 1 diabetes patients (D). * p< 0,05 ** p<0,01.
(TIF)

S7 Fig Frequency of CD4+ Teff activated PD1+ cells relative to HD and type 1 diabetes upon treatment with peptide 3 and subsequent stimulation with anti-CD3/CD28 beads. Upper graphs (**a**,**b**,**c**) show the percentage of CD4+ Teff activated PD1+ cells (**a**), CD4+ Teff activated PD1low cells (**b**), CD4+ Teff activated PD1high cells (**c**) after 4 days of anti-CD3/CD28 stimulation. Lower graphs (**d**,**e**,**f**) show the percentage of CD4+ Teff activated PD1+ cells (**d**), CD4+ Teff activated PD1low cells (**e**), CD4+ Teff activated PD1high cells (**f**) after 6 days of anti-CD3/CD28 stimulation Values correspond to mean frequency ± SEM of 14 healthy controls (HD) and 16 long-term type 1 diabetes patients (D). * p< 0,05.
(TIF)

S1 Table Laboratory, metabolic characteristics, *p53* codon 72 and *PTPN22* genotypes of the LT type 1 diabetes patients recruited for the study. HbA1c (mean glycated hemoglobin) reference value <48 mmol/mol. C-peptide reference 0.80–3.80 ng/mL. Pathological values are indicated in bold. Insulin requirement is expressed as IU/Kg/day with reference range for age of 0.6–1.23 IU/Kg/day. gen = genotype. Molecular analysis of the C1858T (R620W) polymorphism of the autoimmunity predisposing gene *PTPN22* was evaluated using an XcmI restriction fragment length polymorphism-PCR (polymerase chain reaction) method (reviewed in [4]).
(DOCX)

S2 Table Molecular typing for HLA-A, -B, -C, -DRB1 and–DQB1 loci. (DOCX)
