# Supplementary figures and images for: Effect of p53 activation through targeting MDM2/MDM4 heterodimer on T regulatory and effector cells in the peripheral blood of Type 1 diabetes patients
Source: PLoS One. 2020 Jan 29;15(1):e0228296. doi: 10.1371/journal.pone.0228296 (PMC6988923; doi:10.1371/journal.pone.0228296)

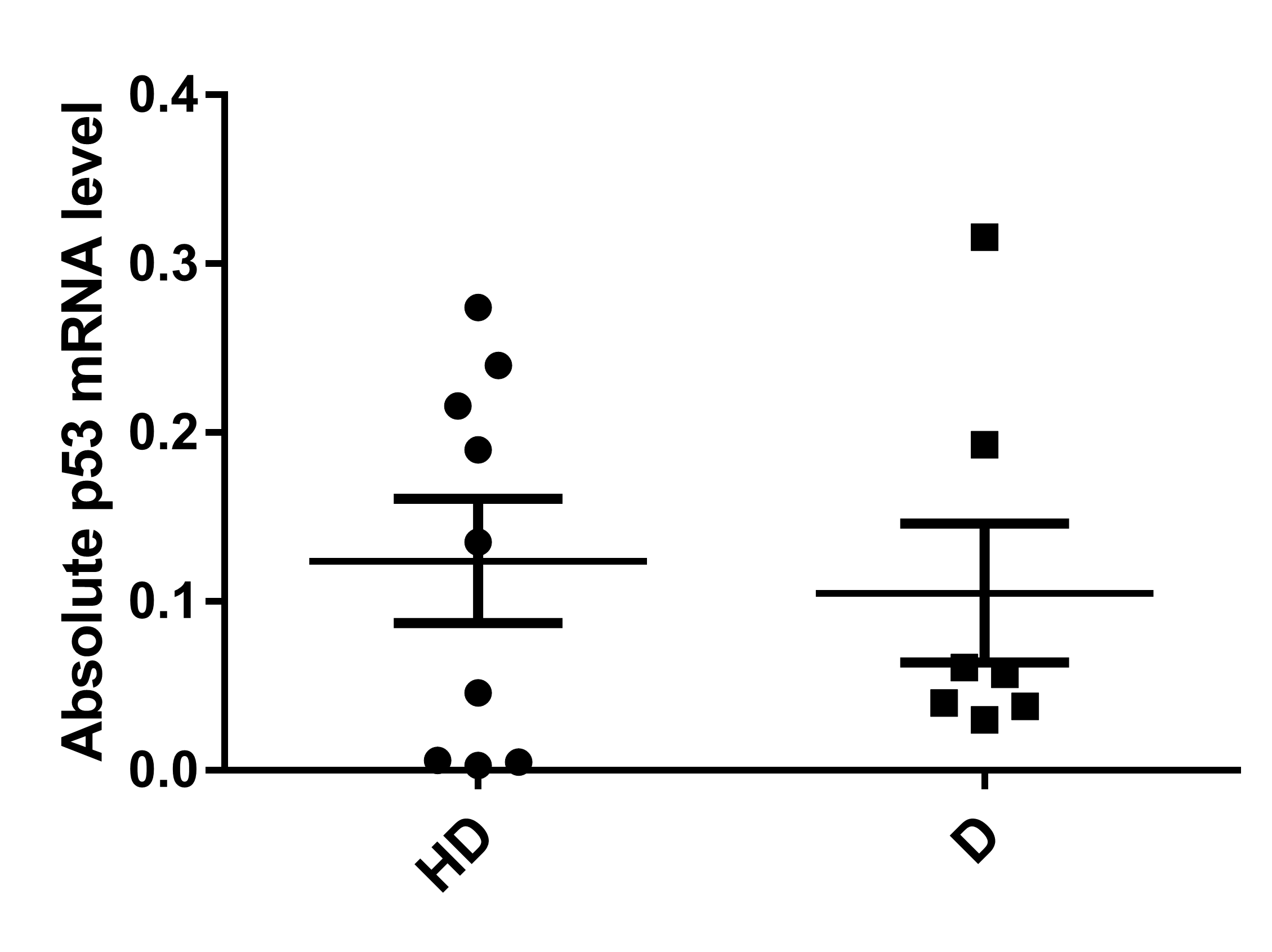

Supplement: S3 Fig — Messenger RNA for p53 in PBMC from 7 LT type 1 diabetic patients and 9 HD controls was quantified by rtq-PCR analysis. Each symbol represents an individual; horizontal lines show the mean ± SEM. p = 0.7377. (TIF) [file pone.0228296.s003.tif]

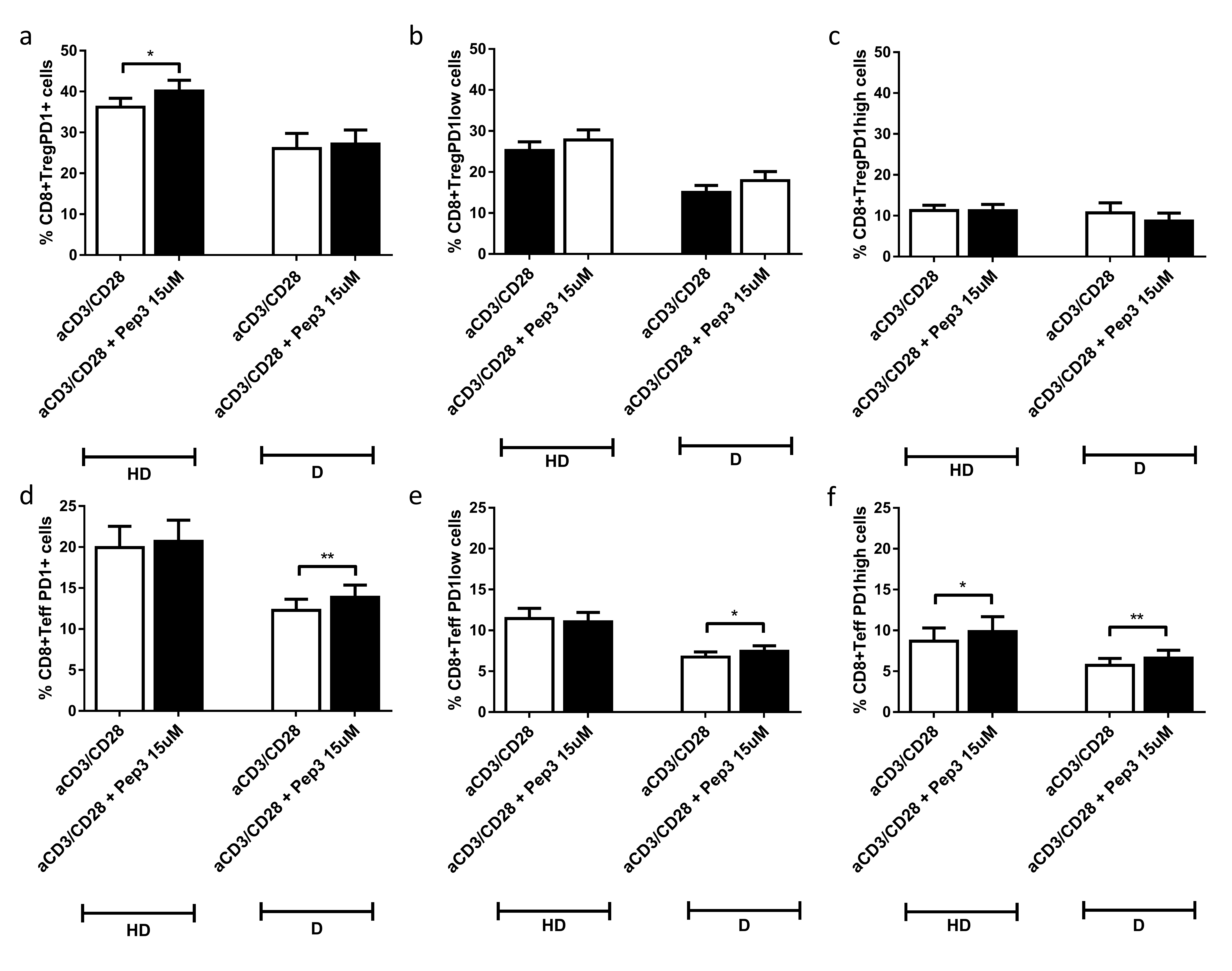

Supplement: S4 Fig — Graphs show the percentage of CD8+ Treg PD1+ cells (a), CD8+ Treg PD1low cells (b), CD8+ Treg PD1high cells (c), CD8+ Teff PD1+ cells (d), CD8+ Teff PD1low cells (e), CD8+ Teff PD1high cells (f). Percentages of PD1+, PD1low and PD1high cells were evaluated in comparison to the corresponding parental subset under evaluation. Values correspond to mean frequency ± SEM of 14 healthy controls (HD) and 16 long-term type 1 diabetes patients (D). * p< 0,05 ** p<0,01. (TIF) [file pone.0228296.s004.tif]

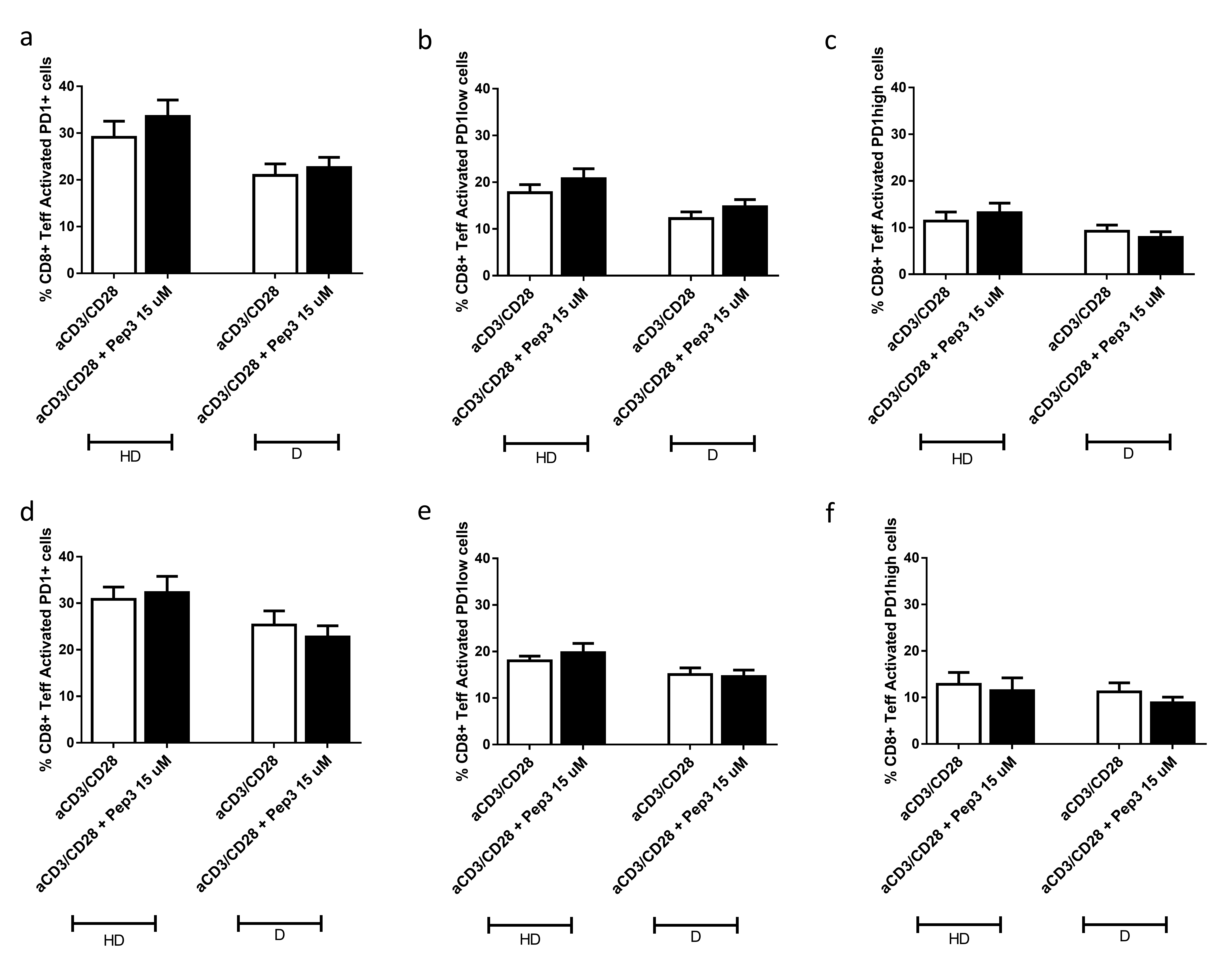

Supplement: S5 Fig — Upper graphs (a,b,c) show the percentage of CD8+ Teff activated PD1+ cells (a), CD8+ Teff activated PD1low cells (b), CD8+ Teff activated PD1high cells (c) after 4 days of anti-CD3/CD28 stimulation. Lower graphs (d,e,f) show the percentage of CD8+ Teff activated PD1+ cells (d), CD8+ Teff activated PD1low cells (e), CD8+ Teff activated PD1high cells (f) after 6 days of anti-CD3/CD28 stimulation Values correspond to mean frequency ± SEM of 14 healthy controls (HD) and 16 long-term type 1 diabetes patients (D). (TIF) [file pone.0228296.s005.tif]

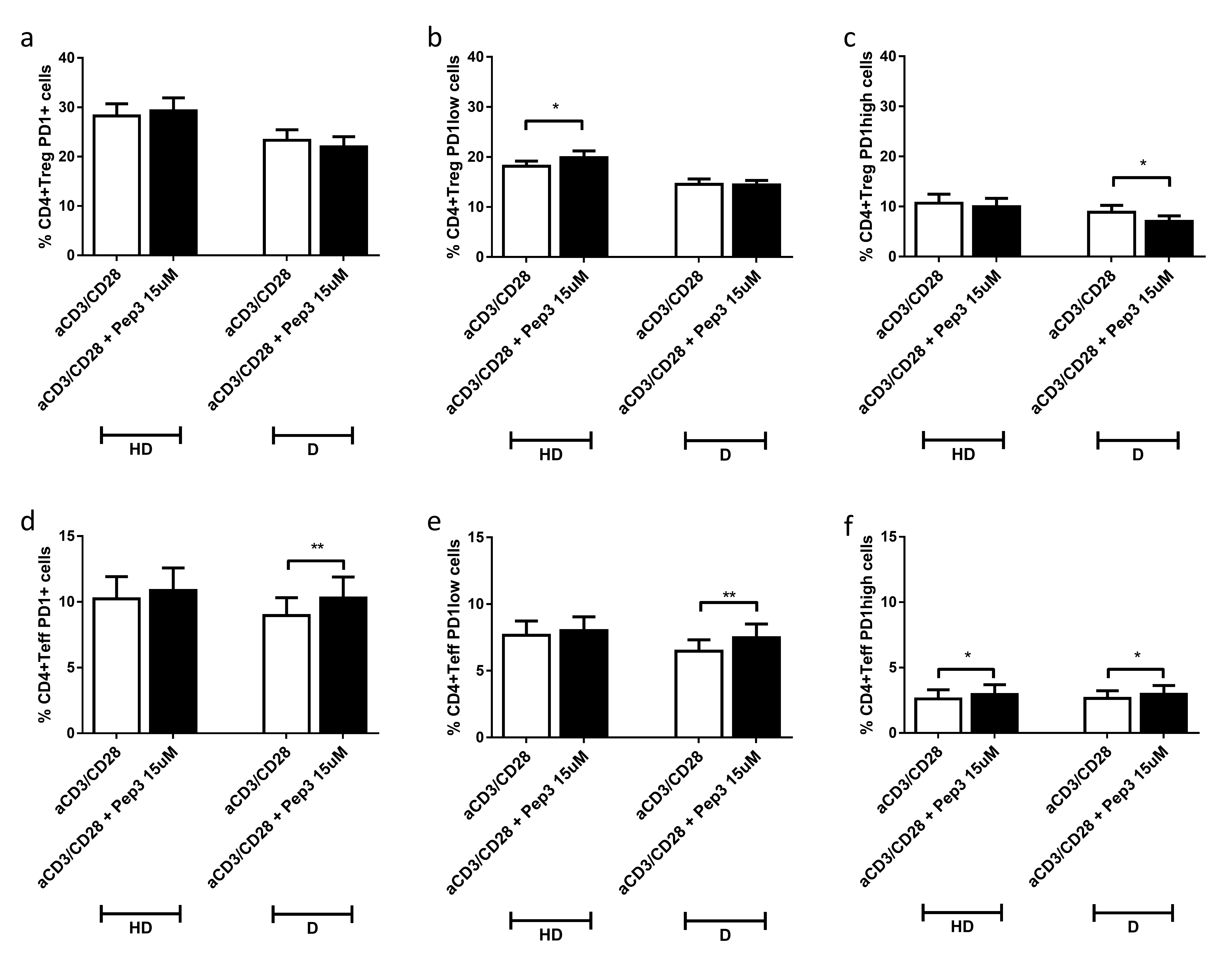

Supplement: S6 Fig — Graphs show the percentage of CD4+ Treg PD1+ cells (a), CD4+ Treg PD1low cells (b), CD4+ Treg PD1high cells (c), CD4+ Teff PD1+ cells (d), CD4+ Teff PD1low cells (e), CD4+ Teff PD1high cells (f). Values correspond to mean frequency ± SEM of 14 healthy controls (HD) and 16 long-term type 1 diabetes patients (D). * p< 0,05 ** p<0,01. (TIF) [file pone.0228296.s006.tif]

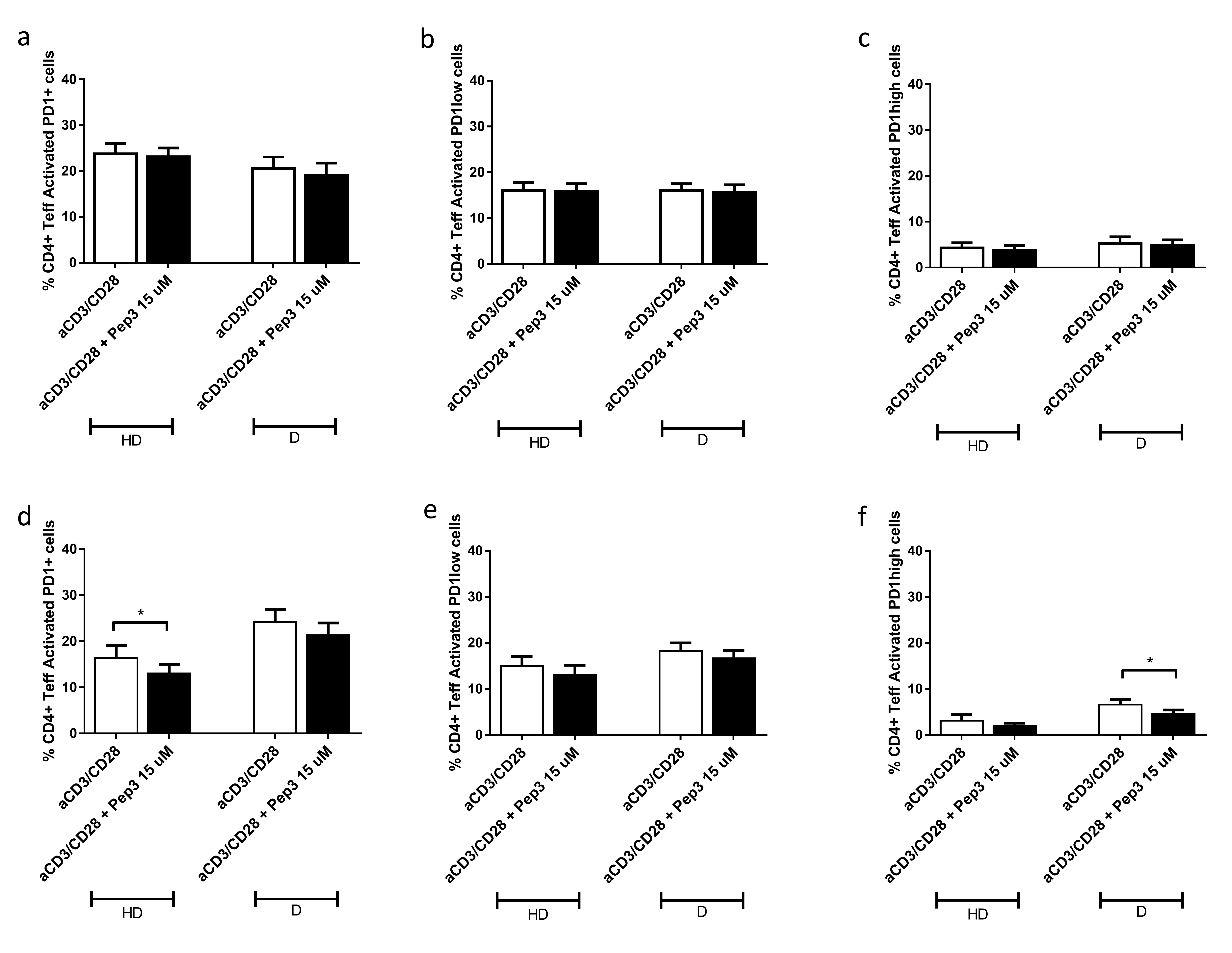

Supplement: S7 Fig — Upper graphs (a,b,c) show the percentage of CD4+ Teff activated PD1+ cells (a), CD4+ Teff activated PD1low cells (b), CD4+ Teff activated PD1high cells (c) after 4 days of anti-CD3/CD28 stimulation. Lower graphs (d,e,f) show the percentage of CD4+ Teff activated PD1+ cells (d), CD4+ Teff activated PD1low cells (e), CD4+ Teff activated PD1high cells (f) after 6 days of anti-CD3/CD28 stimulation Values correspond to mean frequency ± SEM of 14 healthy controls (HD) and 16 long-term type 1 diabetes patients (D). * p< 0,05. (TIF) [file pone.0228296.s007.tif]
