# Supplementary material for: Effect of p53 activation through targeting MDM2/MDM4 heterodimer on T regulatory and effector cells in the peripheral blood of Type 1 diabetes patients
Source: PLoS One. 2020 Jan 29;15(1):e0228296. doi: 10.1371/journal.pone.0228296 (PMC6988923; doi:10.1371/journal.pone.0228296)
Supplement: S1 Table — HbA1c (mean glycated hemoglobin) reference value <48 mmol/mol. C-peptide reference 0.80–3.80 ng/mL. Pathological values are indicated in bold. Insulin requirement is expressed as IU/Kg/day with reference range for age of 0.6–1.23 IU/Kg/day. gen = genotype. Molecular analysis of the C1858T (R620W) polymorphism of the autoimmunity predisposing gene PTPN22 was evaluated using an XcmI restriction fragment length polymorphism-PCR (polymerase chain reaction) method (reviewed in [4]). (DOCX) [file pone.0228296.s008.docx]

| **Pt** | **HbA1c**  **(mmol/mol)** | **c- peptide**  **(ng/mL)** | **insulin**  **requirement**  **(IU/Kg/day)** | **p53**  **codon 72**  **gen** | ***PTPN22***  **gen** |
| --- | --- | --- | --- | --- | --- |
| 1 | **57** | **<0.05** | 0.88 | *Pro/  *Pro | C1858C |
| 2 | **53** | **0.07** | 0.88 | *Pro/  *Pro | C1858C |
| 3 | **64** | **<0.05** | 0.67 | *Pro/  *Pro | C1858C |
| 4 | **73** | **<0.05** | 1 | *Arg/  *Pro | C1858C |
| 5 | **72** | **<0.05** | 0.98 | *Pro/  *Pro | C1858C |
| 6 | **61** | **<0.05** | 0.84 | *Pro/  *Pro | C1858C |
| 7 | **63** | **<0.05** | 0.69 | *Pro/  *Pro | C1858C |
| 8 | **71** | **<0.05** | 0.96 | *Arg/  *Pro | C1858C |
| 9 | 41 | **<0.05** | 0.84 | *Pro/  *Pro | C1858C |
| 10 | **84** | **<0.05** | 0.63 | *Pro/  *Pro | C1858C |
| 11 | **61** | **<0.05** | 0.96 | *Pro/  *Pro | C1858C |
| 12 | **61** | **<0.05** | 0.68 | *Arg/  *Pro | C1858C |
| 13 | 32 | **<0.05** | 0.94 | *Pro/  *Pro | C1858C |
| 14 | **59** | **<0.05** | 0.81 | *Pro/  *Pro | C1858C |
| 15 | **51** | **<0.05** | 0.6 | *Arg/  *Pro | C1858C |
| 16 | **78** | **<0.05** | 1.2 | *Pro/  *Pro | C1858C |

**Supplementary Table 1. Laboratory, metabolic characteristics, *p53* codon 72 and *PTPN22* genotypes of the LT type 1 diabetes patients recruited for the study.** HbA1c (mean glycated hemoglobin) reference value <48 mmol/mol. c-peptide reference 0.80-3.80 ng/mL. Pathological values are indicated in bold. Insulin requirement is expressed as IU/Kg/day with reference range for age of 0.6-1.23 IU/Kg/day. gen = genotype. Molecular analysis of the C1858T (R620W) polymorphism of the autoimmunity predisposing gene *PTPN22* was evaluated using an XcmI restriction fragment length polymorphism-PCR (polymerase chain reaction) method (reviewed in [4]).
