# Supplementary material for: Effect of p53 activation through targeting MDM2/MDM4 heterodimer on T regulatory and effector cells in the peripheral blood of Type 1 diabetes patients
Source: PLoS One. 2020 Jan 29;15(1):e0228296. doi: 10.1371/journal.pone.0228296 (PMC6988923; doi:10.1371/journal.pone.0228296)
Supplement: S2 Table — (DOCX) [file pone.0228296.s009.docx]

| Pt | A | B | C | DRB1 | DQA1 | DQB1 |
| --- | --- | --- | --- | --- | --- | --- |
| 1 | *02:05 | *08:01 | *07:01 | *03:01 | *05:01 | *02:01 |
|  | *24:02 | *49:01 | *07:02 | *04:05 | *03:01 | *03:02 |
| 2 | *01:01 | *07:05 | *15:05 | *03:01 | *05:01 | *02:01 |
|  | *02:01 | *08:01 | *07:02 | *03:01 | *05:01 | *02:01 |
| 3 | *03:01 | *07:05 | *15:05 | *04:05 | *03:01 | *02:01 |
|  | *29:02 | *39:10 | *12:03 | *03:01 | *05:01 | *03:02 |
| 4 | *24:02 | *07:02 | *07:01 | *03:01 | *05:01 | *02:01 |
|  | *29:01 | *44:02 | *05:01 | *04:02 | *03:01 | *03:02 |
| 5 | *24:02 | *15:17 | *07:01 | *03:01 | *05:01 | *02:01 |
|  | *68:02 | *35:01 | *04:01 | *13:02 | *01:02 | *06:04 |
| 6 | *23:01 | *41:01 | *04:01 | *03:01 | *05:01 | *02:01 |
|  | *24:02 | *44:03 | *04:01 | *04:05 | *03:01 | *03:02 |
| 7 | *02:01 | *08:01 | *07:01 | *03:01 | *05:01 | *02:01 |
|  | *02:01 | *15:17 | *07:01 | *03:01 | *05:01 | *02:01 |
| 8 | *03:01 | *07:02 | *07:02 | *08:01 | *04:01 | *04:02 |
|  | *03:05 | *38:01 | *12:03 | *13:02 | *01:02 | *06:04 |
| 9 | *02:01 | *18:01 | *12:03 | *03:01 | *05:01 | *02:01 |
|  | *30:01 | *41:01 | *07:01 | *04:01 | *03:01 | *03:02 |
| 10 | *02:01 | *51:01 | *16:02 | *01:01 | *01:01 | *05:01 |
|  | *24:02 | *56:01 | *01:02 | *01:02 | *01:01 | *05:01 |
| 11 | *11:01 | *18:01 | *05:01 | *03:01 | *05:01 | *02:01 |
|  | *24:02 | *51:01 | *15:02 | *04:04 | *03:01 | *03:02 |
| 12 | *03:01 | *07:05 | *15:05 | *03:01 | *05:01 | *02:01 |
|  | *29:02 | *39:10 | *12:03 | *04:05 | *03:01 | *03:02 |
| 13 | *01:01 | *52:01 | *12:02 | *03:01 | *05:01 | *02:01 |
|  | *24:02 | *57:01 | *06:02 | *04:01 | *03:01 | *03:02 |
| 14 | *02:01 | *35:08 | *15:02 | *04:05 | *03:01 | *02:01 |
|  | *11:01 | *73:01 | *15:05 | *16:01 | *01:02 | *05:02 |
| 15 | *24:02 | *18:01 | *05:01 | *03:01 | *05:01 | *02:01 |
|  | *26:01 | *39:06 | *07:01 | *08:01 | *04:01 | *04:02 |
| 16 | *03:01 | *14:02 | *08:02 | *01:02 | *01:01 | *05:01 |
|  | *29:02 | *35:02 | *04:01 | *11:04 | *05:01 | *03:01 |

**Supplementary Table 2.** Molecular typing for HLA-A, -B, -C, -DRB1 and –DQB1 loci.
